# Involvement of Bacterial and Fungal Extracellular Products in Transformation of Manganese-Bearing Minerals and Its Environmental Impact

**DOI:** 10.3390/ijms24119215

**Published:** 2023-05-24

**Authors:** Bence Farkas, Hana Vojtková, Zuzana Farkas, Domenico Pangallo, Peter Kasak, Antonio Lupini, Hyunjung Kim, Martin Urík, Peter Matúš

**Affiliations:** 1 Institute of Laboratory Research on Geomaterials, Faculty of Natural Sciences, Comenius University in Bratislava, Mlynská dolina, Ilkovičova 6, 84215 Bratislava, Slovakia; bence.farkas@uniba.sk (B.F.); martin.urik@uniba.sk (M.U.); 2 Department of Environmental Engineering, Faculty of Mining and Geology, VŠB–Technical University of Ostrava, 17. Listopadu 15/2172, 708 00 Ostrava, Czech Republic; hana.vojtkova@vsb.cz; 3 Institute of Molecular Biology, Slovak Academy of Sciences, Dúbravská Cesta 21, 84551 Bratislava, Slovakia; zuzana.farkas@savba.sk (Z.F.); domenico.pangallo@savba.sk (D.P.); 4 Center for Advanced Materials, Qatar University, Doha P.O. Box 2713, Qatar; peter.kasak@qu.edu.qa; 5 Department of Agraria, Mediterranea University of Reggio Calabria, Feo di Vito snc, 89124 Reggio Calabria, Italy; antonio.lupini@unirc.it; 6 Department of Earth Resources and Environmental Engineering, Hanyang University, 222 Wangsimni-ro, Seongdong-gu, Seoul 04763, Republic of Korea; kshjkim@hanyang.ac.kr

**Keywords:** manganese, biotransformation, microorganisms, manganese oxides, sorption

## Abstract

Manganese oxides are considered an essential component of natural geochemical barriers due to their redox and sorptive reactivity towards essential and potentially toxic trace elements. Despite the perception that they are in a relatively stable phase, microorganisms can actively alter the prevailing conditions in their microenvironment and initiate the dissolution of minerals, a process that is governed by various direct (enzymatic) or indirect mechanisms. Microorganisms are also capable of precipitating the bioavailable manganese ions via redox transformations into biogenic minerals, including manganese oxides (e.g., low-crystalline birnessite) or oxalates. Microbially mediated transformation influences the (bio)geochemistry of manganese and also the environmental chemistry of elements intimately associated with its oxides. Therefore, the biodeterioration of manganese-bearing phases and the subsequent biologically induced precipitation of new biogenic minerals may inevitably and severely impact the environment. This review highlights and discusses the role of microbially induced or catalyzed processes that affect the transformation of manganese oxides in the environment as relevant to the function of geochemical barriers.

## 1. Introduction

Geochemical barriers are epigenetic zones with diverse functional characteristics related to their distinct physical or chemical gradients in the soil or sediment environments [1]. They can decrease the migration capacity of chemical compounds, and, consequently, due to the accumulation of elements within their bodies, natural ore deposits are formed at these zones [2].

One of the prevailing and vital components of the geochemical barriers are manganese oxides [3], which affect the immobilization of both inorganic and organic compounds due to their significant sorption and redox properties [4,5,6]. They are considered the strongest naturally occurring oxidants [7], and their diverse crystalline forms serve as a pool of essential elements, including manganese. Therefore, their composition and other chemical features may affect the proper functioning of cellular metabolic pathways and the organisms’ physiological state within the geochemical barriers [8,9].

Throughout the history of Earth’s ore formation and mineral diversification, microorganisms have been a driving force in major geological events. For example, the Great Oxidation Event (~2.2 to 2.0 Ga) and the evolution of eukaryotic microorganisms led to direct and indirect biotransformation of an initial ~1500 mineral species, resulting in an increase to over 4000 species [10,11]. Therefore, throughout Earth’s history, autochthonous microorganisms have developed various strategies to transform and acquire manganese and other elements associated with manganese oxides [12,13].

This is usually promoted by the interaction of reactive microbial extracellular metabolites with the surfaces of manganese phases, resulting in the gradual dissolution and transformation of its oxides [14]. These processes are primarily mediated by the redox and protolytic reactions, in which the microorganisms (both bacteria and fungi) may be involved directly or indirectly [15,16]. Consequently, microorganisms possess an exceptional ability to deteriorate and transform the manganese-bearing minerals, thus, altering their reactivity and stability in the environment [17,18,19]. However, the biodeterioration of manganese oxides in natural geochemical barriers may also contribute to releasing associated potentially hazardous elements [3,18,19,20], adversely affecting the environment’s vitality [21].

In addition to the release of hazardous elements, microbially induced biodeterioration of manganese phases supports the development of sustainable agriculture by increasing the bioavailability of various essential nutrients (e.g., phosphorus and nitrogen) [22,23] and bioavailable manganese, which plays a crucial role in various metabolic processes in plants including ROS scavenging and photosynthesis [12,24,25].

From a holistic point of view, the role of microorganisms in optimal or excessive manganese availability for plants should not be overlooked. Manganese deficiency can occur in dry calcareous soils, while its toxicity occurs in poorly drained acidic soils [26,27].

Since we consider the biologically induced transformation of manganese oxides essential for the mobility of both the nutrients and potentially toxic elements, the following review introduces the biogeochemical aspects of manganese oxides’ microbially driven transformation in the natural environment and highlights the capacities of microorganisms to alter these reactive phases by various direct and indirect mechanisms.

## 2. Geochemistry of Manganese in Soils

Manganese can exist in different forms in soils and sediments, including Mn(II), Mn(III), and Mn(IV). This variety of oxidation states leads to numerous manganese minerals in these environments. Post [28] reported that at least thirty different crystal structures of manganese oxides occur in the environment, with the most prevalent being birnessite, vernadite, hollandite, lithiophorite, pyrolusite, todorokite, cryptomelane, hausmannite, and romanechite. Of these, the vernadite and birnessite are the most widespread [29], although the birnessite content can be actually lower than reported in favor of vernadite [30].

In the soil environment, the manganese can be found in soil solution in dissolved form (mostly as complexed Mn(II)); it is adsorbed onto the surfaces of the soil mineral components and soil organic matter or sequestered in organisms. Still, the major pool of soil manganese comprises primary or secondary minerals [31].

The manganese content in the surface soil horizons is very variable. Bowen [32] estimated that the global average manganese concentration is 1000 mg·kg^−1^, ranging from 20 mg·kg^−1^ to 10,000 mg·kg^−1^. The reported value is identical to the concentration of manganese in the lithosphere, which indicates the dependence of the soil manganese on its content in the parent rock [33]. It also suggests that the manganese remains largely immobile during regional metamorphism. Thus, rock-forming minerals are the primary source of manganese in soils. There, manganese is predominantly associated with ferromagnetic silicates since Mn(II) is capable of an isomorphic substitution with Fe(II) [34].

In sediments, the manganese is more prevalent in fine-grained fractions and primarily associates with the layered silicates, sesquioxides, and carbonates, while its content in mature quartzose sandstones is low. Therefore, the soils that are formed from mafic volcanic rocks (with a manganese content over 1000 mg·kg^−1^) or shales rich in iron and magnesium contain higher amounts of manganese in comparison to soils developed from granite or sandstone (up to 400 mg·kg^−1^ Mn) [35].

Manganese is usually bound to minerals in rocks that form under reducing conditions, which predominantly causes Mn(II) to occur in the primary minerals. However, in the weathering zone, the rocks are exposed to water and permanent or fluctuating oxidizing conditions, which allows the incorporation of manganese into the weathering products. Under these conditions, it is oxidized to metastable Mn(III) or to stable Mn(IV), while Mn(II) ion is being leached out by the reactive components from the aqueous solution. From the mentioned naturally occurring manganese species, the metastable Mn(III) drives oxidative activity in organic soil layers [36]. At the same time, the released Mn(II) ion can precipitate to form secondary minerals, e.g., oxides and oxyhydroxides [37], and potentially can form coatings on rock surfaces and mineral particles [28]. Therefore, the redox conditions are one of the dominating factors which control the Mn speciation in the soils. In addition to redox conditions of the soil environment, the prevailing pH is also a determining factor in manganese speciation. Under acidic soil conditions (pH < 5.5) the bioavailable Mn(II) is favored [38], while at a higher pH range, the species of Mn(III) and Mn(IV) are likely. The increase in one pH unit leads to a 100-fold decrease in Mn(II) concentrations [39]. Despite the natural mobilization of manganese (excluding anthropogenic sources), the concentration of its soluble forms in the surface waters only exceeds 1000 µg·L^−1^ in exceptional conditions and usually does not reach the concentration of 200 µg·L^−1^ [40].

The processes of manganese oxides and oxyhydroxides dissolution and precipitation regulate the mobility of manganese in soils and sediments and its availability to organisms. As we mentioned earlier, the solubility of manganese oxides is primarily a function of pH. It decreases in the order of pyrochroite > hausmannite > bixbyite > manganite > birnessite > nsutite > pyrolusite (Table 1). The dissolution of manganese oxides can also be facilitated by the presence of various chelating ligands [41].

**Figure 1:**
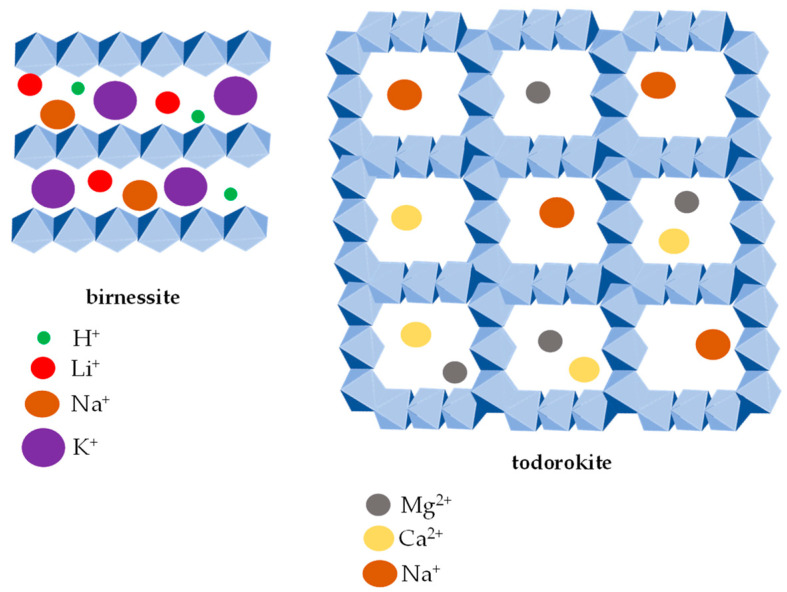
The tunnel- and layer-type crystal structures of Mn oxides. The layer-type crystal structure is represented by birnessite (**left** image) with various interlayer cations H^+^, Li^+^, Na^+^ and K^+^. Todorokite (**right** image) represents the tunnel type (3 × 3) crystal structure with Mg^2+^, Ca^2+^ and Na^+^ cations in the central tunnels.

The stability of manganese oxides in the soil environment depends not only on the redox reactions and pH but also on the crystal structure of the respective mineral. The main types of crystal structures of manganese oxides include tunnel and layered structures [28] whose elementary unit is the MnO_6_ octahedron [3]. The tunnel structure is formed by the MnO_6_ octahedron chains sharing the corners of the neighboring chains, resulting in typical square or rectangular “tunnels” (Figure 1).

Manganese oxides with the layered structure consist of MnO_6_ octahedron-based layers, and birnessite (Figure 1) is a typical representative. Depending on the degree of hydration and the cations’ size, the layered structures may expand or collapse. The typical dimensions of the interlayer spaces are 7 or 10 Å. While the Ca(II), Mg(II), Ni(II) and Cu(II) cations stabilize the structure of 10 Å phyllomanganates, the H(I), K(I), Pb(II), Ce(III) and Th(IV) cations stimulate the collapse of the crystal structure [48]. However, manganese with a tunnel structure (e.g., todorokite) (Figure 1) does not collapse or expand [49].

The layered structures of manganese oxides are the precursors of tunnel-structured oxides [50]. The conversion between these is possible in soils, and it depends on the temperature [51], the ratio of Mn(III) to Mn(IV) [52], pH [53], light conditions [54] and the nature of the present cation between the layers of the precursors. For example, Mg(II) saturated precursors transform into todorokite [55] while Na(I) saturated precursors convert to synthetic cryptomelane [56].

## 3. Transformation of Manganese by Microorganisms

As we mentioned in the previous chapter, the transformations of manganese to various species and their environmental abundance are significantly influenced by the prevailing pH and redox conditions. Except for these abiotic determinants, several biotic factors affect the geochemistry of manganese, among which the microorganisms play an essential role.

Microorganisms are capable of modifying and maintaining the distinctive pH [57], and redox conditions [58,59] in their microenvironment due to their metabolic [60] and enzymatic activities [61], which enables the processes of manganese-bearing minerals’ and dissolved species’ transformations. These include (i) the oxidation of dissolved Mn(II) that results in the precipitation of Mn(III), Mn(IV) or mixed-valence oxides, (ii) the reduction of insoluble manganese oxides into the mobilizable Mn(II), and (iii) the chelation of dissolved Mn(II) with organic exudates that triggers the formation of biogenic minerals [62,63,64,65]. Furthermore, some microorganisms can oxidize and reduce manganese simultaneously, e.g., *Bacillus pumilus* and *B. cereus* [66].

### 3.1. Manganese Oxidation by Bacteria

The microbial oxidation of Mn(II) in the presence of dissolved or free oxygen will manifest in a way that is likely to follow the mechanism of abiotic (Equation (1)) transformation:

Mn^2+^ + H_2_O + 0.5O_2_ ↔ MnO_2_ + 2H^+^ (1)

Although the oxidation of Mn(II) to Mn(III) or Mn(IV) states is thermodynamically favored, especially at pH values and partial pressures that are characteristic for the upper soil horizons and the surface waters (e.g., pH 6.5 to 8.5 and pO_2_ ~ 21 kPa), the kinetics of the process are extremely slow under such environmental conditions [67]. However, the presence of microorganisms can significantly increase the manganese transformation rate [68]. Biological oxidation of Mn(II) through Mn(III) to Mn(IV) is characteristic for various phylogenetic bacterial groups, e.g., the species and isolates belonging to Firmicutes (*Bacillus subtilis*, *Bacillus* sp. MB-11), Actinobacteria (*Arthrobacter globiformis*) or Proteobacteria (*Leptothrix discophora*, *Erythrobacter* sp.) [3].

**Figure 2:**
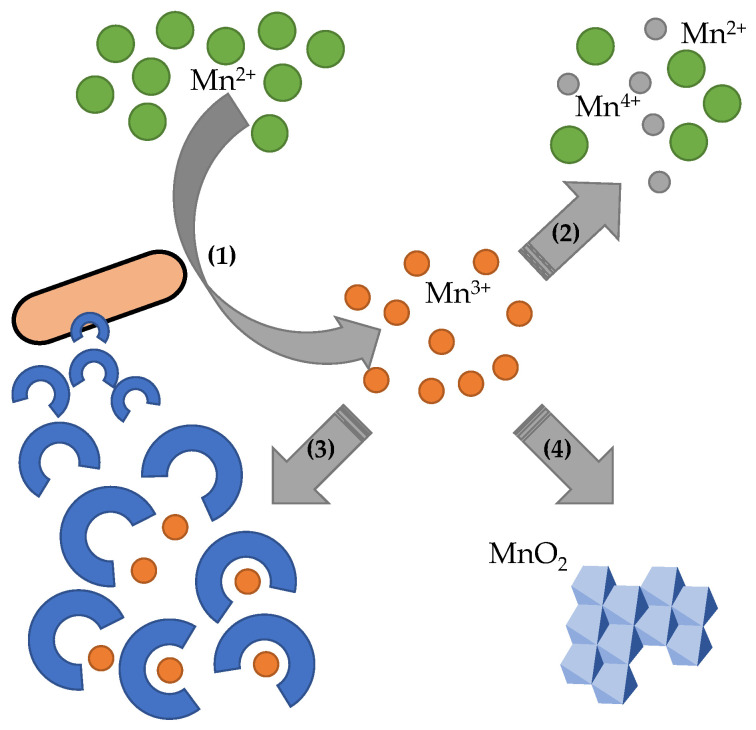
Schematic illustration of manganese oxidation by bacteria. (1) Enzymatic oxidation of dissolved manganese results in the formation of metastable Mn(III), which readily (2) disproportionates into Mn^2+^ and Mn^4+^. Bacterially produced secondary metabolites, e.g., bacterial pyoverdines, (3) stabilize the metastable Mn(III), which makes the additional oxidation of Mn(III) possible and leads to (4) MnO_2_ precipitation.

The molecular mechanism of biologically induced manganese oxidation has been studied in the Gram-positive bacterium *Bacillus* sp. SG-1 [69]. This model organism oxidizes Mn(II) using a multi-copper oxidase (MCO) MnxG that is localized in the exosporium of bacterial spores [70]. The redox transformation of Cu(II)/Cu(I) in MnxG enables the electron exchange, which leads to effective regulation of manganese as well as homeostasis of other metals both directly and indirectly in the cell (Figure 2) [71]. The regulation is primarily due to the formation of an insoluble layer around bacterial spores comprising Mn(IV) oxides with high sorptive and redox capacities [72]. Furthermore, due to the formation of the insoluble biogenic Mn(IV) oxides, not only the Mn(II) is transformed, but the biogeochemical cycles of various elements in the surrounding environment are also affected [73].

It is apparent that Mn(II) oxidation to Mn(IV) requires a two-electron transfer. However, the MnxG catalyzes only the transfer of one electron. Therefore, it was assumed that MnxG catalyzes the transformation of Mn(II) to Mn(III), followed by another bio-catalyzed one-electron transfer. The experimental data have confirmed that this is actually a two-step process since during the bacterial Mn(II) oxidation of a metastable Mn(III) has been identified (Figure 2) [74]. In the kinetic studies of manganese oxidation, a pyrophosphate was used as a stabilizing agent for Mn(III) in the solution. The formed Mn(III) complex with pyrophosphate was determinable by UV-Vis spectrometry [75]. In the presence of microorganisms, the metastable Mn(III) is most likely stabilized via complexation with the extracellular metabolites for a period sufficient for Mn(III) to be oxidized by MCO [76,77].

Interestingly, the formation of Mn(III) complexes has an important influence on iron acquisition. For example, iron deficiency in strains of *Pseudomonas putida* MnB1 and GB-1 led to the production of the fluorescent pyoverdine (a siderophore) that can form a stable complex with Fe(III). However, the extruded pyoverdines bound Mn(III) more efficiently than Fe(III); thus, the competitive relationship prevents the formation of manganese oxides and restricts the uptake of iron by bacteria [78,79].

Siderophores also promote the oxidation of Mn(II) indirectly by providing a negative charge from the ligand to Mn(II), which reduces the activation energy of oxidation and promotes the electron transfer from Mn(II) to O_2_ [63,80]. Biogenic siderophores are common in surface waters [13,81] and in soils [82], and thereby, their role in Mn(II) oxidation in the environment is relevant.

Another indirect process of Mn(II) oxidation is a production of an inorganic oxidizing agent by microorganisms. The strain of *Leptothrix pseudoochraceae* produces H_2_O_2_ during aerobic growth by metabolizing glucose and other organic substrates. This facilitates the oxidation of Mn(II) and precipitation of MnO_2_. While this process is not important in terms of energy metabolism, it helps microorganisms reduce the elevated concentrations of toxic H_2_O_2_ [83].

### 3.2. Oxidation of Manganese by Filamentous Fungi

In the previous section, we introduced the microbial oxidation process of Mn(II) by bacteria. Unfortunately, manganese oxidation by microscopic filamentous fungi is significantly less studied, although they are expected to play a similar role in the manganese biogeochemistry as the bacteria [62].

The white rot fungi (mostly of *Basidiomycota* division), which can degrade lignin and certain aromatic pollutants [84] via the activity of manganese peroxidase [85], are the most studied group of Mn(II) oxidizing fungi. The manganese peroxidase transforms the Mn(II) to Mn(III) by a single-electron transfer [86]. Synthesized Mn(III) is then chelated by the fungal exudates, degrading lignin phenolic units. Otherwise, the Mn(III) ions are disproportionate to manganese oxides and Mn(II) [87].

The ascomycetous fungi are also able to break down lignin, however, the mechanism of degradation is not identical to white rot fungi since it is not manganese dependent, and they preferentially degrade carbohydrates [88]. The oxidation of Mn(II) by ascomycetous fungi does not serve any apparent physiological benefit for this fungal group; thus, its utilization is in contrast to some bacterial strains that are capable of conserving energy during the oxidation of Mn(II) to MnO_2_ by coupling the oxidation to ATP synthesis [89,90]. In the case of fungi, the Mn(II) oxidation is not linked to energy conservation [91] and neither the growth or the cell differentiation of fungus is enhanced in the presence of Mn(II).

**Figure 3:**
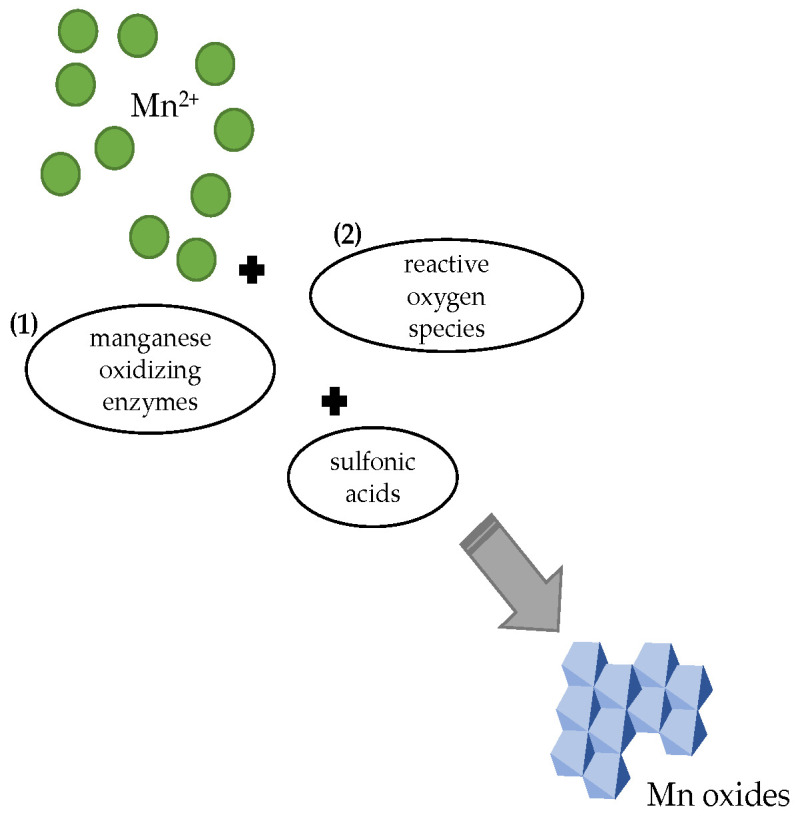
Oxidation of manganese by the filamentous fungus may occur (1) directly by activity of enzymes, or (2) indirectly via extracellularly produced reactive oxygen species (e.g., superoxide). The oxidation process can be mitigated by supplementation of organosulfur compounds (sulfonic acids).

The *Ascomycetes* are capable of producing the reactive oxygen species (ROS), including the superoxide (O_2_^−^) diradical [92], which is a key redox oxidant that plays a significant role in the geochemical transformation of numerous metals, including the oxidation of Mn(II) (Figure 3) [84,93]. Since the abiotic oxidation of Mn(II) is unfavorable under pH 8 in oxic environments, the oxidation of Mn(II) in surface waters is usually slow. This is due to thermodynamically unfavorable electron transfer from Mn(II) to molecular oxygen. However, the superoxide is more likely to gain an electron from Mn(II) [94].

In the fungal kingdom, the extracellular production of superoxide is widespread [95], and it is involved in hyphal branching, cell signaling, and cell differentiation [96]. The NADPH-oxidases are responsible for the superoxide production in fungi. Hansel et al. [84] studied the interaction of soluble Mn(II) during the cultivation of the filamentous fungus *Stilbella aciculosa*. The presence of Mn(II) resulted in the formation of a brown precipitate deposited at the base of reproductive structures. The X-ray absorption spectroscopy confirmed that the manganese associated with the fungus was predominantly in the form of Mn(IV) (80%), while the abundance of Mn(III) (11%) and Mn(II) (9%) was lesser. The precipitated phase consisted primarily of birnessite, a hydrous manganese dioxide mineral that belongs to the dominant biogenic manganese oxides formed by microorganisms [97]. Biogenic birnessite is a highly disordered mineral with a high degree of layer site vacancies. Due to these characteristics, it possesses high sorptive and oxidative capacities. Therefore, it seems that microorganisms purposely oxidize Mn(II) to deposit birnessite outside the cell, which can then act as a protection layer against the toxic metals, or it enables the oxidation of recalcitrant organic compounds to increase the pool of organic carbon sources [98].

Hansel et al. [84] noted that the precipitation of the manganese oxides in the adjacent space of the hyphae and conidiophore of *S. aciculosa* is likely an accidental side reaction related to the exudation of extracellular superoxide since the superoxide production is linked to cell differentiation of the fungus. Still, this is an interesting homology between fungal and bacterial Mn(II) oxidation mechanisms since some bacterial oxidation of Mn(II) is also mediated by superoxide, such as the case of bacterial strain *Roseobacter* Azw-3b [99,100].

Since the Mn(II) oxidation by fungi is superoxide dependent, the reactive oxygen species (ROS) scavengers generally inhibit the formation of manganese oxide. Superoxide dismutase (SOD) [99] and Cu(II) [101] are considered effective deteriorators of superoxide. The production of superoxide in fungi serves as a signal for the proliferation of asexual and sexual reproductive structures [102]; therefore, by increasing the Cu(II) concentration over 100 µM, not only the Mn(II) oxidation is suppressed, the formation of reproductive structures is also inhibited [96].

According to a study by Zeiner et al. [100], the Mn(II) oxidation by *Ascomycetes* could be also catalyzed enzymatically by various species of *Ascomycetes* (*Stagonospora* sp., *Pyrenochaeta* sp., and *Paraconiothyrium sporulosum*), which are capable of producing the Mn(II) oxidizing enzymes, including the glucose–methanol–choline (GMC) oxidoreductases, tyrosinase, bilirubin oxidase, and glyoxal oxidase (Figure 3) [100].

Manganese oxides found in the environment are predominantly biogenic, formed by the oxidation of Mn(II) through biological processes by various microorganisms [3,62]. The biosynthesized Mn oxides are diverse, as various Mn(II) oxidation mechanisms occur. The large diversity of biogenic Mn oxides depends on the synthesizing microorganism (e.g., bacteria or fungus). Still, the prevailing conditions during biosynthesis also have significant relevance. For example, the microscopic filamentous fungus *Acremonium* sp. produced two types of manganese oxides during the distinguished cultivation conditions. When the cultivation resulted in cell suspension, the δ-MnO_2_ was formed. However, the surface-attached growth of the fungus induced the δ-MnO_2_ and todorokite formation [103]. Moreover, the Mn(II)-oxidative capacity of various groups of microorganisms can vary based on their metabolic activity, e.g., the acidogenic group has less oxidative capacity in comparison to the non-acidogenic group. Furthermore, the increase in glucose concentration supports the rate of Mn(II) oxidation by the acidogenic species [104].

Hinkle et al. [105] reported that the sulfonic acids, i.e., the organosulfur compounds, could influence the Mn(II) oxidizing ability of various ascomycetous fungi since they have promoted the Mn(II) oxidation by *Plectosphaerella cucumerina* DS2psM2a2 and resulted in a formation of hexagonal birnessite. In the case of *Paraphaeosphaeria sporulosa* AP3s5–JAC2a, the oxidation to Mn(IV) was suppressed by the supplemented sulfonic acids and were observed during the cultivation formation of biogenic bixbyite (Mn_2_O_3_). Meanwhile, the supplementation of sulfonic acids exerted a minimal effect on Mn(II) oxidation by *Stagonospora* sp. SRC1lsM3a [105].

### 3.3. Bacterial Reduction of Manganese

**Figure 4:**
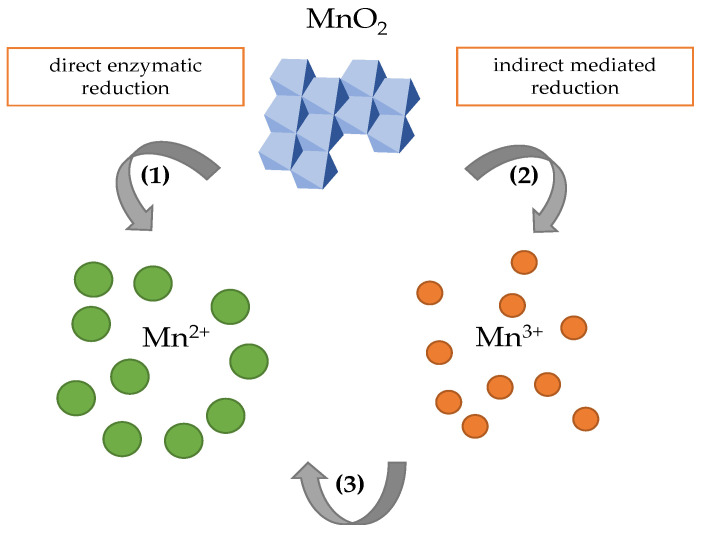
Bacterial reduction of Mn oxides by (1) extracellular reductases directly reduces Mn oxides through a one-step process. (2) Indirect reduction occurs in a two-step process, which is driven by secondary extracellular metabolites (e.g., oxalate, sulfite) where (3) an intermediate Mn(III) is formed in the first step and reduced in the second step.

Microbially induced manganese reduction has an important role in geochemical processes occurring in natural waters, aquifers, and soil systems [106,107]. The reduction of manganese oxides by microorganisms can be indirect (a non-enzymatic transformation) or direct (mediated by the extracellular reductases) (Figure 4). During the indirect reduction, the produced metabolites serve as reducing agents, e.g., formic acid, pyruvate, sulfite or oxalate [15].

The mechanism of biocatalyzed reduction of Mn(IV) is considered as a one-step process where two electrons are transferred, and the final product of the reaction is Mn(II) [16]. The indirect reduction by reducing agents [108] is considered a two-step process where the transfer of one electron to MnO_2_ takes place, and the metastable intermediate product of Mn(III) is formed (Figure 4) [109].

Since Mn(IV) is prevalent in amorphous and crystalline oxides and oxyhydroxides under neutral soil conditions, it is relatively complicated for the microorganisms to utilize direct (enzymatic) reduction of these complex phases [110]. Therefore, microorganisms capable of transforming the Mn(IV) imply various extracellular ligands to extract manganese from the manganese precipitates by complexolysis in order to increase its bioavailability. The formed Mn(IV) organometallic compounds are then transformed by reductases in the periplasmic space of bacteria [111]. A different strategy is to use an electron transfer via an endogenous or exogenous compound, which is at first enzymatically reduced and then used as a reducing agent during the indirect redox transformation of Mn(IV) [112].

The reduction of manganese in higher valency states has an important role in the energy metabolism of some microorganisms (e.g., *Alteromonas putrefaciens* MR-1) that use Mn(IV) as a terminal electron acceptor [113] during the oxidation of organic compounds or hydrogen. At the same time, the bacterial strain *Sulfurimonas marisnigri*, which was isolated from the Black Sea, showed a capacity to couple the reduction of MnO_2_ to the oxidation of H_2_S or thiosulfate for energy generation [114]. Furthermore, the strain of *S. marisnigri* was able to completely reduce MnO_2_ towards the end of the growth phase, and the reduced Mn(II) precipitated into carbonates.

It was reported that the growth of *Shewanella oneidensis* coincided with the reduction of Mn(III) to Mn(II) [109], which highlighted the significance of Mn(III) intermediate in bacterial metabolism and geochemistry of sulfur and manganese in sea sediments [115]. Manganese intermediate is also key in acetate oxidation in aquatic environments [116]. Szeinbaum, et al. [117] reported that marine bacteria *Shewanella* sp. can couple anaerobic acetate oxidation with Mn(III) reduction. In this case, the acetate consumption and Mn(III) reduction has been in a ratio of from 1 to 6, which means that, for one acetate molecule, six Mn(III) ions have been reduced. However, the ratio was lower than theoretical values since acetate has been partially used in endergonic metabolic pathways [117]. Still, the process has been predominantly exergonic and relates to organic carbon mineralization [115].

The formation of the Mn(III) intermediate is generally either driven via the reductive dissolution of Mn(IV) oxides by siderophore-like ligands [118] or is synthesized during microbial Mn(II) oxidation [74]. The dissolved Mn(III) is metastable, and it is therefore necessary to stabilize it in the aqueous solution with ligands complexation, including humic substances [119] and inorganic pyrophosphate [120]. The Mn(III)-complexes can still donate or accept the electrons; therefore, they can act as both the reductant and oxidant [119]. Thus, in the case of Mn(III) as an extracellular electron acceptor, the large abundance of Mn(III) at oxic and anoxic interfaces [121] can support microbial activity and preserve the microbial populations until the point when a more appropriate substrate for growth becomes available [117].

### 3.4. Reduction of Manganese-Bearing Rocks and Minerals by Filamentous Fungi

**Figure 5:**
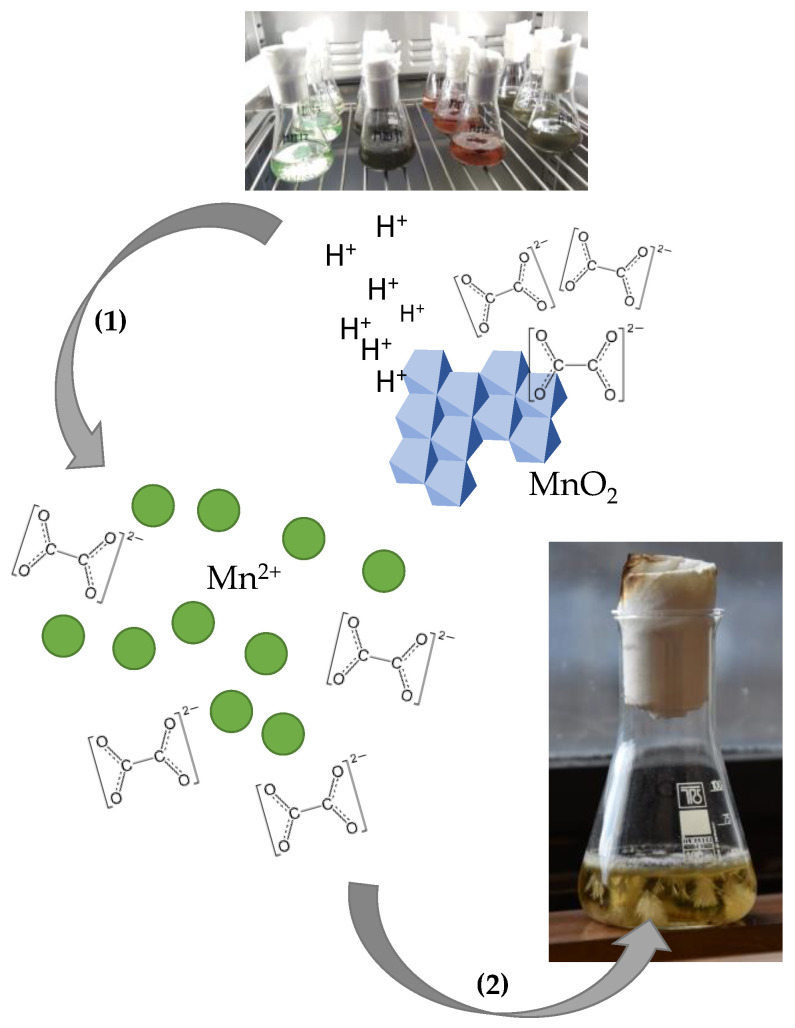
Reduction of Mn oxides by microscopic filamentous fungus. (1) Extracellular metabolites mediate the Mn oxides’ biodeterioration and dissolution via acidolysis, redoxolysis, and complexolysis. Accumulated secondary metabolic products with chelating properties (e.g., oxalate) form complexes with dissolved manganese. This may result in (2) precipitation of stable biogenic minerals (e.g., manganese oxalate).

Filamentous fungi can also reduce the manganese minerals or manganese-bearing rocks. However, they do not benefit from the reduction of manganese of higher valences compared to bacteria since the fungal energy metabolism is not associated with the manganese. Therefore, the main reason for manganese reduction in fungi is nutrition acquisition. Thus, there is also a difference in the prevalent mechanism of manganese reduction by filamentous fungi compared to other microorganisms. Fungi mainly reduce the Mn(IV) indirectly (non-enzymatically) by producing extracellular metabolites that act as reductants [15]. The indirect reduction of Mn(IV) leads to the solubilization of Mn(IV) bearing minerals and the formation of a soluble metal-ligand complex (Figure 5) [122].

The organic metabolites that are linked to the process of Mn(IV) reduction and dissolution [123] are predominantly the low-molecular-weight extracellular organic acids that have exceptional chelating and redox properties [124]. Heterotrophic fungi synthesize these (e.g., gluconic, oxalic, citric, and acetic acids) by conversion of the primary sugar sources [125]. However, it is important to note that fungi usually excrete these in dissociated form since the intracellular pH is neutral [126]. However, other than the production of chelating organic ions, the coinciding extrusion of H^+^ which facilitates the Mn(IV) reduction is due to mineral or rock dissolution (Figure 5) [127].

Acharya, et al. [128] identified two extracellular metabolites, oxalate and citrate, that are responsible for the bioextraction of manganese from manganese ore by the fungus *Penicillium citrinum*. The fungal strain was able to extract approximately 68% of manganese from the ore during 45-day cultivation. In comparison, the 0.5 M sulfuric acid was able to extract only up to 1.2% of manganese in 30 days, which is considered a negligible efficiency. Srimekanond, et al. [129] noted that the mineralogy of the ore may play a prominent role in microbial community bioleaching performance. The organic carbon pools are also important factors that affect efficient manganese reduction and extraction. Acharya, et al. [130] noted a direct relation between the manganese extraction efficiency of manganese ore and sugar concentration in the media. They noted that the maximum extent of manganese bioextraction was 33% in the case of 10% sucrose concentration while the lowest sucrose content (2%) reached only 7% of manganese bioextraction efficiency. The authors hypothesized that the absence of sufficient amounts of organic carbon substrates resulted in a decrease in the extracellular metabolites’ exudation, and, thus, the fungus *P. citrinum* was unable to extract manganese from the ore sufficiently.

Since acidolysis can be an alternative or co-occurring pathway of manganese oxides’ deterioration [131], the acidification of culture media facilitates the manganese oxides’ dissolution [17]. Still, acidification is only partially responsible for the manganese bioextraction and plays a secondary role compared to reductive dissolution [128]. Godunov, et al. [132] also reported that the diluted sulfuric acid solutions were not capable of dissolving Mn_2_O_3_ and Mn_3_O_4_ completely. This is due to the formation of the thermodynamically stable MnO_2_ on the surfaces of the initial oxides (Equations (2) and (3)). However, if oxalate (Equation (4)) is supplemented to the sulfuric acid solution, the dissolution rate is considerably accelerated [133].

Mn_2_O_3_ + 2H^+^ ↔ Mn^2+^ + MnO_2_ + H_2_O (2)

Mn_3_O_4_ + 4H^+^ ↔ 2Mn^2+^ + MnO_2_ + 2H_2_O (3)

Xyla et al. [131] hypothesized that the accelerated effect of oxalate on MnO_2_ deterioration in acidic media was due to formation of surface oxalate-MnO_2_ complex that resulted in an electron transfer and a release of the reduced Mn(II) into the solution. Thus, during the dissolution, oxalate, Mn(IV)-oxide, and protons are consumed (Equation (4)) as follows:

MnO_2_ (s) + C_2_O_4_ ^2−^ + 4H^+^ ↔ Mn^2+^ + 2CO_2_ + 2H_2_O (4)

Other than the aforementioned factors, the extent of Mn(II) extraction from the manganese-bearing minerals and ores by filamentous fungi is influenced by various other factors, such as pulp density of manganese mineral and composition of manganese ore, and temperature. The strain of *Aspergillus niger* managed to extract 91% of manganese from oceanic polymetallic nodules (5% *w/v*) [134], 69% from the synthetic Mn_3_O_4_ [17] and 78.8% from the low-grade pyrolusite [135]. Fungus *Aspergillus oryzae* reached the maximum of 79% extraction efficiency under optimized conditions (pH 6, 37 °C, 2% pulp density of manganese ore and dextrose as carbon source) [125].

Furthermore, when evaluating the manganese extraction efficiency, we also need to take into account the readsorption of Mn(II) ions onto the solid manganese phases [136], as well as the precipitation of biogenic mineral phases [17]. In a culture medium, the elevated concentration of Mn(II) and accumulated extracellular oxalate allows the formation of secondary minerals, including manganese oxalate trihydrate (falottaite) and manganese oxalate dihydrate (lindbergite) [65,137]. However, the formation of manganese oxalate phases can be beneficial for the producing microorganism. The elevated dissolved concentrations of Mn could have inhibiting effects towards microbial growth, and therefore, complexation of Mn onto oxalate phases possess indirect detoxification effects/outcomes in the case of the fungus *A. niger* [17]. In addition to the favorable outcomes for microorganisms, the formation of oxalate phases also influences the geochemistry of some potentially toxic elements, e.g., arsenic [65].

## 4. The Role of Manganese in Geochemical Barriers

**Figure 6:**
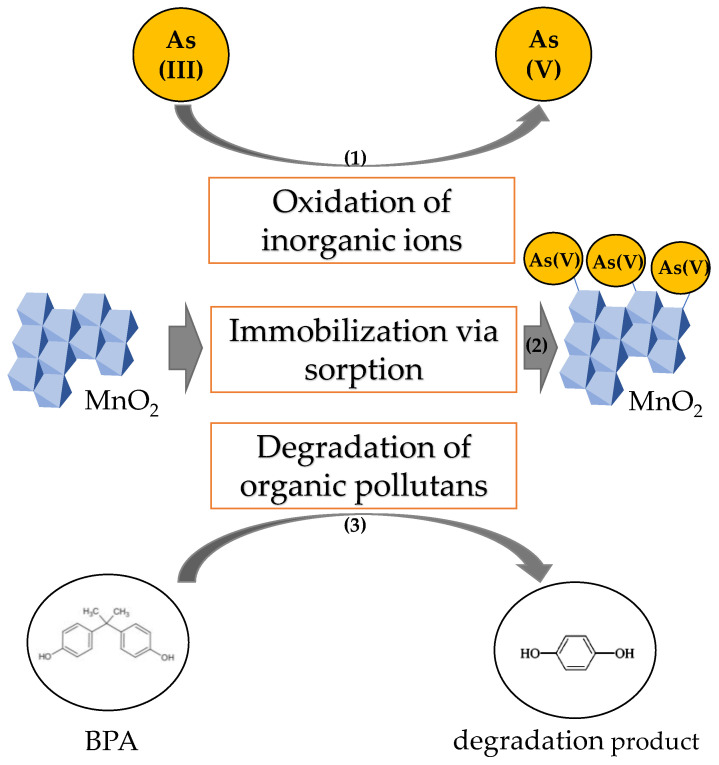
The schematic illustration of processes that are governed by Mn oxides in geochemical barriers. There, Mn oxides can (1) transform, (2) adsorb and (3) degrade potentially harmful organic or inorganic pollutants.

Due to their redox and adsorption capacities, manganese minerals (primarily oxides and oxyhydroxides) control the speciation and mobility of various compounds in soil, freshwater, and marine sediments [138]. Furthermore, these natural scavengers excel in the adsorption of ions, thereby contributing to the immobilization of various metals and metalloids [139,140,141]. Their exceptional reactivity can be competed only with iron oxides. Furthermore, manganese oxides are reportedly capable of oxidizing a wide range of organic pollutants, including azo compounds [142], hormones [143] and antibiotics (Figure 6) [144]. As a result, synthetic and natural (hydrated) manganese oxides and oxyhydroxides are applied as active components in the engineered geochemical barriers for immobilization and transformation of organic and inorganic contaminants.

The reaction pathway of phenolic compounds’ oxidation includes electron transfer from phenolic groups to manganese oxides, leading to phenoxyl radical formation. The radicals undergo rearrangement, and manganese oxides could further oxidize more stable intermediates [145]. Thus, the transformation of organic compounds can lead to a depletion of manganese oxides of higher valences, and the concentration of dissolved Mn(II) increases simultaneously [146].

Decontamination of the persistent chemical warfare [147] and organophosphate pesticides [148] is also doable via transformation in the presence of manganese oxides. Mesoporous adsorbents based on MnO_2_ nanobelts showed promising results in decontaminating chemical warfare, such as sarin, sulfur mustard, and chloroethyl sulfide. The MnO_2_-based nanobelts could decompose these compounds by forming non-toxic products [147].

In the case of bisphenol A, an omnipresent environmental contaminant with endocrine disruption potential, the manganese oxides have been shown capable of oxidation in aqueous solutions [149]. However, in the soil environment, the transformation of phenolic compounds can be inhibited by soil pH [150] or the presence of other organic compounds, e.g., dissolved organic matter [151].

Manganese oxides have also shown great potential to remediate sites contaminated with inorganic substances, e.g., the labile and toxic As(III) can be oxidized to a more stable and less toxic As(V) in their presence. The kinetics of As(III) oxidation under natural conditions are very slow and it can take several months for As(III) to be oxidized completely. However, manganese oxides can decrease the oxidation half-life of As(III) oxidation to 10–20 min (Figure 6) [152], which seems beneficial for the ecosystem. On the other hand, the oxidation of inorganic metals or metalloids by manganese oxides can also have adverse effects on the environment. This is the case with chromium, which is more toxic in its hexavalent form. Therefore, reducing Cr(VI) to Cr(III) is preferential in the remediation processes. However, it was reported that Cr(III) is susceptible to oxidation and form Cr(VI) in the presence of birnessite [153], thus, manganese oxides are not utilizable in the remediation of media contaminated with Cr(VI).

Other than the ability of manganese oxides to oxidatively transform the organic and inorganic compounds into less toxic or, in some cases, potentially harmful substances, manganese oxides also possess the excellent capability to immobilize these transformants. Furthermore, due to their specific structure and surface, manganese oxides are effective adsorbents of wide range of metals and metalloids, including As, Sb, Se, Hg, Cu, Co, Pb, Zn, and Cd [3,154,155,156]. Thus, in the case of arsenic, the manganese oxides play a role in remediation processes as a redox-active component of the geochemical barrier and provide sorption sites for both As(III) and As(V) species. However, it is important to note that the ferric oxides seem more suitable for arsenic immobilization. Thus, the application of a two-step treatment is a suitable solution for the remediation of arsenic-contaminated media, starting with As(III) oxidation by manganese oxides that is followed by As(V) adsorption onto ferric oxides [140,157]. Still, in some cases, ions are more efficiently adsorbed onto manganese oxides compared to ferric oxides [158].

Xie, et al. [159] noted that manganese oxides are promising reactive components for removing Se (IV) from contaminated environments. Both predominant selenium species in aqueous environments and soils, oxyanions of Se(IV) and Se(VI), are highly mobile [160], with the former being more toxic for aquatic organisms [161]. Novel research studied the adsorption properties of synthetic δ-MnO_2_ towards Se(IV) and described a formation of inner layer surface complexation of Se(IV) by the manganese phase. Therefore, the Se(IV)-O-Mn(III) complex represented mainly the adsorption process. Moreover, it was observed that, during the adsorption-redox processes, the oxidation of Se(IV) to Se(VI) was coupled to δ-MnO_2_ reduction and Mn(III) was formed as the primary product. As we mentioned earlier, the formed Mn(III) is metastable, and therefore, it is disproportionated to Mn(II) and Mn(IV). The Mn(III) could also be adsorbed onto δ-MnO_2_ or complexed by the microorganisms and act as an enhancer of microbial activity [162]. Nevertheless, the experiments performed by Li et al. [162] showed that Mn(II) adsorption onto the δ-MnO_2_ did not affect the potential sorption positions for Se(IV).

The crystal structure of manganese phases also influences the sorption properties. The presence of internal reactive sites in the layered structure of birnessite caused the increase in the sorption efficiency for Pb(II), while the tunneled structure of cryptomelane (K(Mn^4+^,Mn^2+^)_8_O_16_) possessed a lower sorption capacity [163]. Some cations can occupy limited structural sorption sites. While the substantial immobilization of Pb(II) by the birnessite can be explained by the occupation of both interlayer and surface sites, other cations, including Cu(II), Zn(II), and Cd(II), are preferentially adsorbed onto the interlayer sites [164].

An additional factor that plays a significant role in the cation adsorption affinity towards the surfaces of manganese minerals is the point of zero charge (PZC). Several types of manganese oxides (e.g., cryptomelane, pyrolusite, birnessite) have PZC at pH below 4.7. The variabilities in the value of PZC contribute to the significant differences in the uptake of ions in a wide range of pH between various minerals [165].

Manganese oxides are not the only manganese-bearing minerals in geochemical barriers. Carbonates and biosynthesized organic manganese oxalates also occur in such environments. Recent research reported that the microbially synthesized manganese oxalate phases by the fungus *Aspergillus niger* represent secondary biominerals derived from the kutnohorite (CaMn^2+^(CO_3_)_2_) and todorokite [166,167]. The initial Mn-bearing minerals were transformed into lindbergite (MnC_2_O_4_.2H_2_O) and falottaite (MnC_2_O_4_.3H_2_O), with the latter being less stable and, therefore, being transformed into the more thermodynamically stable dihydrate phase towards the end of cultivation [137,166].

The secondary manganese minerals possess altered sorptive properties, e.g., the transformation of the mineral phase of hausmannite (Mn_3_O_4_) to biomineral lindbergite, which resulted in a decrease of the immobilization efficiency towards Sb(III) [168]. However, the formation of biogenic oxalates had a beneficial effect on As(V) immobilization [65], where the mobility of the potentially toxic element of arsenic decreased, and therefore, the As immobilization by biogenic phases was enhanced compared to the initial phase of manganese oxide [65].

## 5. Concluding Remarks

The main objective of this review is to elucidate the impact of microorganisms (bacteria and fungi) on the stability and environmental fate of manganese oxides. We have highlighted the role of manganese-bearing minerals in geochemical barriers and the influence of naturally occurring biological transformations on the geochemistry of hazardous elements immobilized in these reactive zones. Since microbial consortia can dynamically influence the prevailing conditions in their microenvironment, they also possess the ability to transform stable and insoluble manganese oxides. Some microorganisms chemically deteriorate manganese mineral phases intentionally and, thus, benefit from the transformation energetically, while other microbial groups alter the manganese phases to increase the bioavailability of essential elements associated with their surfaces, which possess excellent sorptive capacities. This is of specific interest to environmental chemists, as microbially mediated manganese oxide transformation, either direct or indirect, may affect the fate and bioavailability of associated potentially toxic elements and trace elements. Furthermore, the biologically induced processes of bioextraction and biotransformation are considered innovative methods for wastewater treatment, the development of sustainable fertilizers in agriculture, the degradation and removal of persistent organic compounds, and the recovery of some industrially important metals from wastes.

However, the implementation of these processes, their optimization, and improvement in efficiency are challenging issues that can be addressed only after their environmental impact and geochemical consequences are known and well understood. 

## Figures and Tables

**Table 1:** Common manganese oxide minerals occurring in soils.

Mineral	PZC	Chemical Formula	Structure
Birnessite	1.18–2.8 [42,43,44]	Na_7_Ca_3_Mn_7_O_14_ 2.8H_2_O	layer
Cryptomelane	1.98–2.1 [42,43]	K_x_(Mn^III^Mn^IV^)_8_O_16_ (x = 1.3–1.5)	tunnel
Hollandite (α-MnO_2_)	4.6 [45]	Ba_x_(Mn^III^Mn^IV^)_8_O_16_(x ˂ 1)	tunnel
Lithiophorite	6.9 [44]	LiAl_2_(Mn^III^Mn^IV^)_3_O_6_(OH)_6_	layer
Todorokite	3.2–3.98 [42,43,44]	(Ca, Na, K)_0.3–0.5_(Mn^III^Mn^IV^)_6_O_12_ 3.5 H_2_O	tunnel
Vernadite (δ-MnO_2_)	2.8–3.1 [46,47]	MnO_2_	layer

## Data Availability

Not applicable.
